# In Consideration of Our Mutual Relationship with Cats

**DOI:** 10.3201/eid2612.AC2612

**Published:** 2020-12

**Authors:** Byron Breedlove, Jana Igunma

**Affiliations:** Centers for Disease Control and Prevention, Atlanta, Georgia, USA (B. Breedlove); British Library, London, UK (J. Igunma)

**Keywords:** art science connection, emerging infectious diseases, art and medicine, about the cover, public health, cats, Felis catus, felines, veterinary medicine, viruses, bacteria, parasites, in consideration of our mutual relationship with cats, Tamrā maeo―Cat treatise, Campylobacter infection, cat–scratch disease, cryptosporidiosis, hookworm infection, rabies, salmonellosis, toxoplasmosis, zoonoses, British Library

**Figure:**
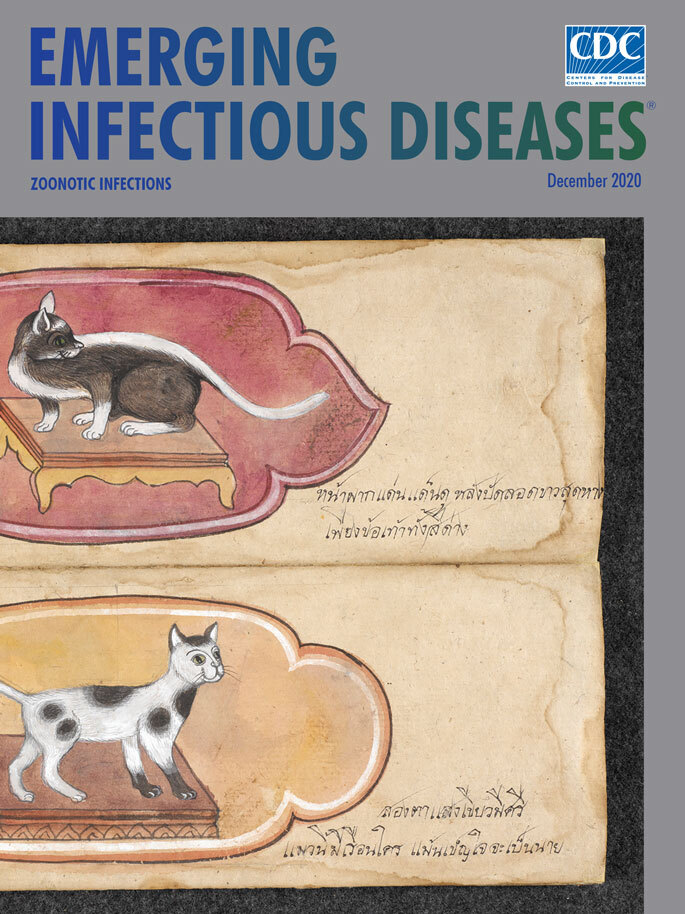
**Artist Unknown. Tamrā maeo―Cat treatise, 1800–1870.** Paper folding book, 12 folios, 2 images per side, ink, Thai script. 14.2 in × 4.7 in/27.31 cm × 28.57 cm. British Library (Or 16797), London, UK. Public domain.

*Felis catus,* the only domesticated species of cat in the family *Felidae*, flourishes on every continent except Antarctica. Able to thrive in almost any climate and habitat, it among the world's most invasive species. Current estimates of the global cat population, including pet, stray, and feral cats, range from 200 million to 600 million. Where there are humans, more than likely there are also cats. 

Humans living in agricultural villages in northern Africa and the Near East are believed to have domesticated the African wildcat (*Felis lybica) *between 8,000 to 12,000 years ago. Archaeologist Magdalena Krajcarz and colleagues noted, “The cat’s way to domestication is a complex and still unresolved topic with many questions concerning the chronology of its dispersal with agricultural societies and the nature of its evolving relationship with humans.” Likely stored grains and trash piles in villages attracted rodent pests, which in turn lured local wildcats and initiated a nascent mutualistic relationship that has since flourished.

From those villages, cats found their way around the world. Authors Lee Harper and Joyce L. White wrote that ancient sailors “were quick to see the advantage of having cats aboard ship during long voyages to protect their food supplies from damage by rodents.” Trade and commerce helped spread cats from the Middle East to various ports of call in Europe, the Far East and Orient, and the Americas. Throughout this common history, cats have been both reviled and revered by humans.

During the Middle Ages in Europe, some religious institutions considered cats evil, leading to thousands being killed. Later, however, the Black Death spread by fleas on rats contributed to cats’ redemption. Harper and White noted, “The cat’s skill as a hunter of vermin was desperately needed. Its reputation was salvaged. Owning a cat was back in style.” 

Ancient Egyptians ascribed to cats many characteristics shared with deities they worshipped. Freyja, Norse goddess of love, beauty, and fertility, rode on a cat-drawn chariot. Temples in medieval Japan often kept a pair of cats to protect precious manuscripts from being ruined by mice. In the Kingdom of Siam, which is modern-day Thailand, Buddhist monks welcomed cats into their temples, where they were protected as *Maeo Wat* (Temple Cats).

This month’s cover art, “two lucky cats to support leadership,” is the second folio from *A Thai Treatise on Cats*, created in the 19th century in central Thailand and acquired by the British Library in 2011. Such manuscripts about cats were made for breeders in Thailand at least from the 18th century on, although it is believed that cat breeding goes back to the beginnings of the ancient Thai Kingdom of Ayutthaya in the 14th century.

This work comprises 12 folios that open from top to bottom to reveal illustrations of 23 different types of Thai cats with brief captions. Author and researcher Martin R. Clutterbuck notes that this Thai treatise (and similar ones) ascribes 18 of these cats to be “lucky” and to bring their owners good luck, rank, prosperity, or health. The remaining cats were deemed unlucky. As is often the case with manuscripts from this period in Thailand, the author or illustrator is not identified nor is this example dated.

The cat depicted at the top is called *Wilat* (Beauty) and described as "having green eyes and dark fur, except the legs, stomach, back and tail which are white." The other cat is named *Kao Taem* (Nine Points) and has shiny emerald eyes and a white coat with nine darker spots of fur. Both males and females make a strong, melodious "*maeo*" sound, and keeping this cat "is understood to result in leadership.” (The names for the different cats in the booklet are commonly used by cat breeders and owners in Thailand.) All cats in this manuscript are depicted on a small pedestal, which is a symbol of respect―deities, monks, royals, and sacred white elephants are often also depicted on pedestals. 

Siamese cats, called *Maeo Boran* in Thai, have a wide variety of coat colors resulting from tyrosinase (TYR) mutations, and their eyes range from blue and green to yellow and gold. Naturally attached and loyal to humans, Maeo Boran are easy to keep in the home and to train to walk on a leash; they often have a beautiful, chatty voice and behave as if they were engaging in lively conversations. 

The positive side of cat ownership, as celebrated in those cat treatises, is acknowledged on the CDC website, “Research has shown that cats can provide emotional support, improve moods, and contribute to the overall morale of their owners. Cats are also credited with promoting socialization among older individuals and physically or mentally disabled people.” Cats, as noted earlier, have also historically helped control the spread of rodent-borne diseases among humans. 

Nonetheless, living in close quarters with cats carries some health risks. Cats can transfer various zoonotic diseases, including* Campylobacter* infection, cat–scratch disease, cryptosporidiosis, hookworm infection, plague, rabies, and salmonellosis. Cats are the only animal in which the *Toxoplasma gondii* parasite completes its life cycle, and humans in close contact with cat litter, for example, are at risk of developing toxoplasmosis, which pregnant women can potentially transmit to a fetus. Much less common is transfer of disease from humans to animals, such as the suspected case of human-to-cat transmission of severe acute respiratory syndrome coronavirus 2 reported in this issue. (Both owner and cat recovered.)

Detecting, responding to, and preparing for emerging zoonotic infections―which, like cats, have made their way around the world with our help―are major challenges for public health leaders. Even if cats are not actual talismans or have the power to improve leadership, spending a few minutes considering these lucky cats may provide public health officials a brief respite or serendipitous insight.
